# Supplementary material for: Revealing carbon capture chemistry with 17-oxygen NMR spectroscopy
Source: Nat Commun. 2022 Dec 15;13:7763. doi: 10.1038/s41467-022-35254-w (PMC9755136; doi:10.1038/s41467-022-35254-w)
Supplement: Supplementary file 1 — Supplementary Information [file 41467_2022_35254_MOESM1_ESM.pdf]

Supplementary Information

Revealing Carbon Capture Chemistry with 17-Oxygen NMR Spectroscopy

Berge, Pugh et al.

Supplementary Table 1. – Overview of the amine-functionalised frameworks investigated in the DFT calculations together with the studied CO_2 adsorption classifications. Unless alternate metals are listed, it is assumed that the Mg framework, *i.e.,* Mg_2(dobpdc), is functionalised with the amine shown. (chain = ammonium carbamate chain, pairs = carbamic acid pairs, mixed = mixed ammonium carbamate – carbamic acid). Unsymmetrical amines are assumed to form a metal-nitrogen bond with the less sterically hindered amine, and also to react with CO_2 at that same amine. Two (R,R)-dach compounds are listed as this chiral diamine interacts with the chiral M_2(dobpdc) framework to form two distinct enantiomers.[3] One tetraamine-functionalised framework was considered, known as 3-4-3–Mg_2(dobpdc).[1]

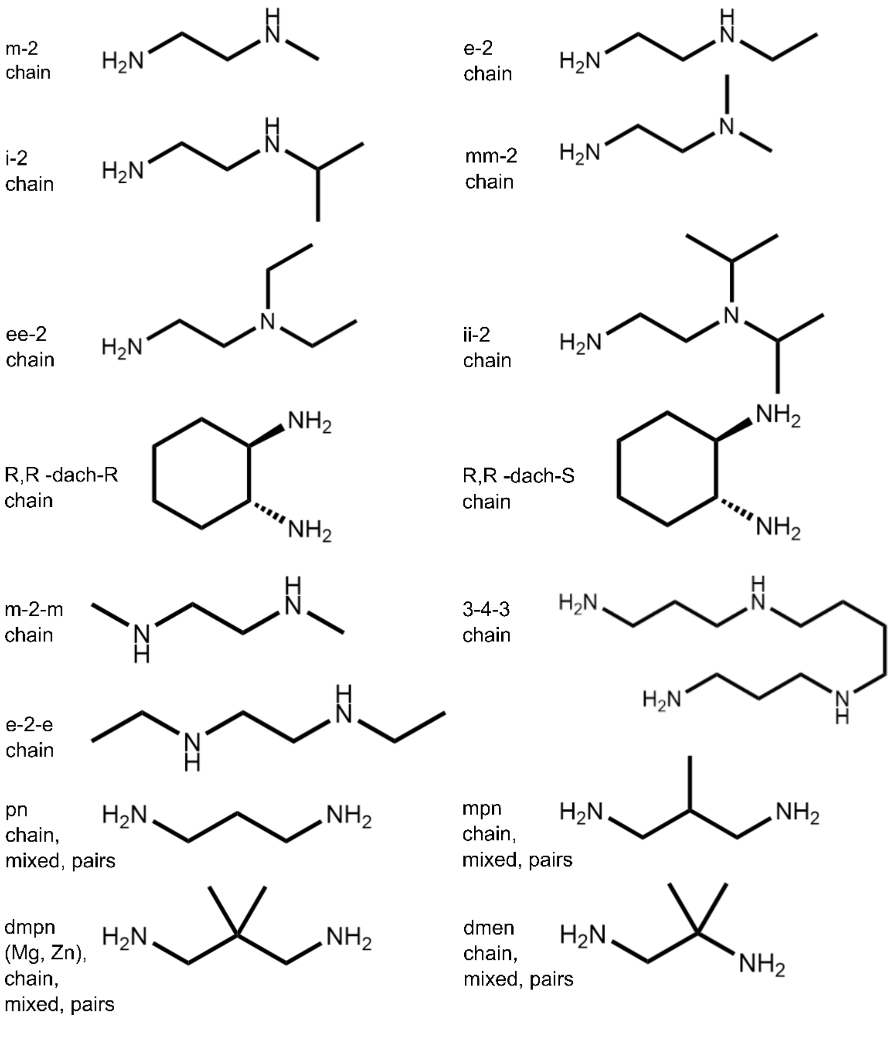

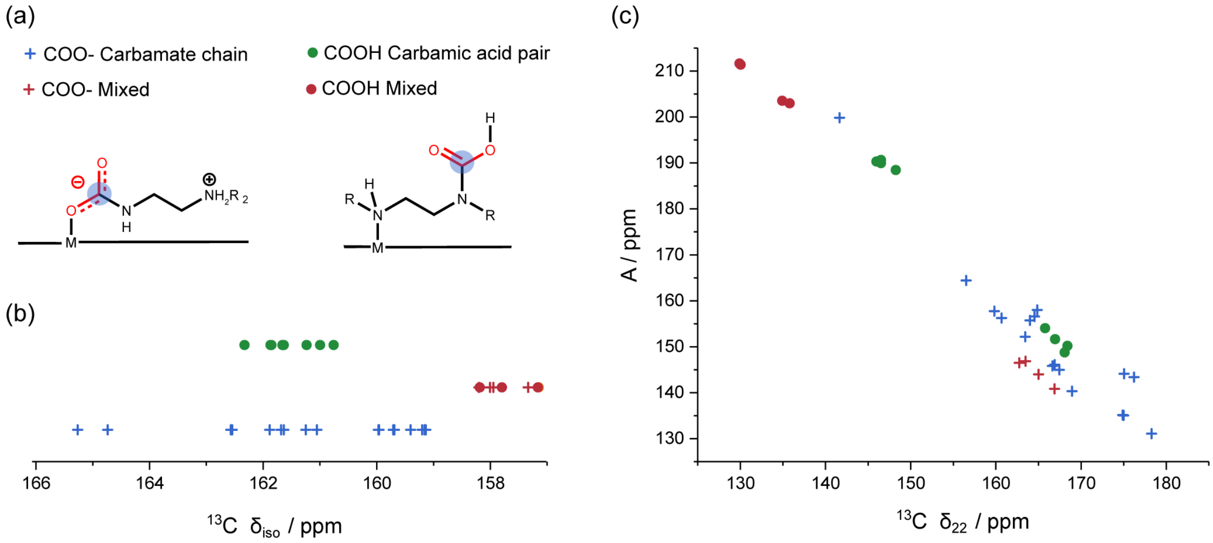

Supplementary Figure 1. – Differentiation of adsorption modes based on ^{13}C NMR. a) Illustrations showing the different carbon environments present in the different CO_2 adsorption structures. b) An overview of the DFT-calculated ^{13}C isotropic chemical shifts for the different classifications showing no clear differentiation between ammonium carbamate chains and carbamic acids. The chemical shift anisotropy of the DFT-calculated ^{13}C NMR shift was also investigated. A plot of $A = (\delta_{11} + \delta_{33} - \delta_{22})$ vs δ_{22} has been shown to differentiate protonated and deprotonated forms of CO_2 adsorbed in amine functionalised silica materials.[4] For our materials, less of a clear differentiation is seen (Figure S1c).

Supplementary Table 2a. – The ^{13}C NMR parameters obtained from DFT calculations for ammonium carbamate chain structures.

*structure at full CO_2 capacity[1]
**structure at half CO_2 capacity[1]
***This structure formed a chain structure with the inserted CO_2 being protonated.

Compound	δ_{iso} (ppm)	δ_{11} (ppm)	δ_{22} (ppm)	δ_{33} (ppm)	Compound	δ_{iso} (ppm)	δ_{11} (ppm)	δ_{22} (ppm)	δ_{33} (ppm)
e-2-Mg_2(dobpdc)	159.97	204.68	166.90	108.33	m-2-Mg_2(dobpdc)	159.40	202.84	168.94	106.43
ee-2-Mg_2(dobpdc)	159.96	206.80	167.45	105.62	i-2-Mg_2(dobpdc)	161.05	231.63	141.66	109.88
mm-2-Mg_2(dobpdc)	159.71	206.76	166.64	105.73	m2m-Mg_2(dobpdc)	162.57	212.85	164.85	110.01
dmen-Mg_2(dobpdc)	161.88	214.75	164.53	106.37	pn-Mg_2(dobpdc)	165.27	215.03	176.21	104.56
mpn-Mg_2(dobpdc)	164.74	214.58	175.04	104.59	dmpn--Mg_2(dobpdc)	159.20	208.87	160.67	108.06
dmpn-Zn_2(dobpdc)	159.14	208.16	159.82	109.43	(R,R)dach-R-Mg_2(dobpdc)	159.16	210.95	156.51	110.01
(R,R)dach-S-Mg_2(dobpdc)	159.69	207.18	163.44	108.44	e-2-e-Mg_2(dobpdc)	161.25	209.05	164.00	110.71
343-Mg_2(dobpdc)*	161.68	203.62	174.99	106.44	343-Mg_2(dobpdc)**	162.54	204.19	178.27	105.17
e2***	159.41	215.30	158.69	104.24					

Supplementary Table 2b. - The ^{13}C parameters obtained from DFT calculations on mixed adsorption structures. For each structure there are two ^{13}C environments.

*This structure formed an alternative mixed structure consisting of one chain of ammonium carbamates, and one chain of carbamic acids.

Compound	Environment	δ_{iso} (ppm)	δ_{11} (ppm)	δ_{22} (ppm)	δ_{33} (ppm)
pn-Mg_2(dobpdc)	M-OCO	157.95	203.25	163.49	107.10
	COOH	157.16	231.31	130.05	110.13
mpn-Mg_2(dobpdc)	M-OCO	157.33	202.51	162.75	106.74
	COOH	157.15	231.83	129.90	109.71
dmpn-Mg_2(dobpdc)	M-OCO	158.20	201.08	166.88	106.65
	COOH	157.80	232.72	134.92	105.75
dmpn-Zn_2(dobpdc)	M-OCO	158.01	201.17	165.01	107.84
	COOH	158.19	231.23	135.79	107.53
ii2-Mg_2(dobpdc)*	M-OCO	162.85	214.15	166.15	108.24
	M-OCOH	158.92	215.67	157.03	104.07

Supplementary Table 2c. - The ^{13}C parameters obtained from DFT calculations on carbamic acid pair structures. The carbamic acid pairs show two different carbon environments. This is due to them being unsymmetric with respect to the organic linker backbone. In this table COOH[a] refers to the part of the pair with the C=O bond pointing towards the backbone. The COOH[b] structure refers to the part of the pair with the C-OH pointing towards the backbone.

Compound	Environment	δ_{iso} (ppm)	δ_{11} (ppm)	δ_{22} (ppm)	δ_{33} (ppm)
pn-Mg_2(dobpdc)	COOH[a]	161.85	214.58	166.95	104.03
pn-Mg_2(dobpdc)	COOH[b]	161.00	228.83	146.52	107.63
mpn-Mg_2(dobpdc)	COOH[a]	161.64	213.46	168.07	103.37
mpn-Mg_2(dobpdc)	COOH[b]	160.76	228.91	145.98	107.39
dmpn-Mg_2(dobpdc)	COOH[a]	161.87	216.68	165.78	103.15
dmpn-Mg_2(dobpdc)	COOH[b]	161.23	230.39	146.52	106.79
dmpn-Zn_2(dobpdc)	COOH[a]	162.33	215.30	168.40	103.29
dmpn-Zn_2(dobpdc)	COOH[b]	161.66	229.99	148.26	106.73

Supplementary Table 3a. - The ^{17}O NMR parameters obtained from DFT calculations on ammonium carbamate chain structures.

*structure at full CO_2 capacity[1]
**structure at half CO_2 capacity[1]
***This structure formed a chain structure with the inserted CO_2 being protonated.

Bold letters are used to indicate the oxygen investigated.

Compound	Site	δ_{iso} (ppm)	C_Q (MHz)	η_Q	Compound	Site	δ_{iso} (ppm)	C_Q (MHz)	η_Q
e-2-Mg_2(dobpdc)	M-**O**CO<br>M-OC**O**	174.09<br>175.05	-7.30<br>-7.25	0.91<br>0.68	m-2-Mg_2(dobpdc)	M-**O**CO<br>M-OC**O**	176.03<br>171.00	-7.31<br>-7.32	0.96<br>0.67
ee-2-Mg_2(dobpdc)	M-**O**CO<br>M-OC**O**	169.10<br>185.47	-7.35<br>-6.97	0.95<br>0.72	i-2-Mg_2(dobpdc)	M-**O**CO<br>M-OC**O**	168.78<br>224.16	-7.56<br>7.94	0.43<br>0.50
mm-2-Mg_2(dobpdc)	M-**O**CO<br>M-OC**O**	177.14<br>164.95	-7.37<br>-7.15	0.93<br>0.66	m-2-m-Mg_2(dobpdc)	M-**O**CO<br>M-OC**O**	151.18<br>194.47	-7.64<br>-6.92	0.97<br>0.88
dmen-Mg_2(dobpdc)	M-**O**CO<br>M-OC**O**	160.17<br>202.07	7.68<br>6.48	0.96<br>0.91	pn-Mg_2(dobpdc)	M-**O**CO<br>M-OC**O**	158.73<br>195.46	7.75<br>6.74	0.73<br>0.84
mpn-Mg_2(dobpdc)	M-**O**CO<br>M-OC**O**	161.76<br>199.02	7.64<br>6.73	0.88<br>0.91	dmpn-Mg_2(dobpdc)	M-**O**CO<br>M-OC**O**	172.59<br>192.79	-7.63<br>-6.93	0.94<br>0.98
dmpn-Zn_2(dobpdc)	M-**O**CO<br>M-OC**O**	170.73<br>192.46	-8.28<br>-6.90	0.91<br>0.95	e2e-Mg_2(dobpdc)	M-**O**CO<br>M-OC**O**	161.16<br>200.56	-7.61<br>-7.04	0.93<br>0.98
343-Mg_2(dobpdc)*	M-**O**CO<br>M-OC**O**	164.73<br>183.41	7.52<br>-7.11	0.84<br>0.72	343-Mg_2(dobpdc)**	M-**O**CO<br>M-OC**O**	170.73<br>188.99	-7.47<br>-7.14	0.87<br>0.85
e2-Mg_2(dobpdc)***	M-**O**COH<br>M-OC**OH**	175.74<br>135.75	7.26<br>-7.94	0.98<br>0.37					

Supplementary Table 3b. - The ^{17}O NMR parameters obtained from DFT calculations on mixed adsorption structures.

*This structure formed an alternative mixed structure consisting of one chain of ammonium carbamates, and one chain of carbamic acids.

Bold letters are used to indicate the oxygen investigated.

Compound	Environment	δ_{iso} (ppm)	C_Q (MHz)	η_Q	Compound	Environment	δ_{iso} (ppm)	C_Q (MHz)	η_Q
pn-Mg_2(dobpdc)	M-**O**CO	168.20	-7.57	0.98	mpn-Mg_2(dobpdc)	M-**O**CO	168.15	-7.54	0.98
	M-OC**O**	181.21	-7.23	0.97		M-OC**O**	182.32	-7.24	0.96
	C**O**OH	220.59	7.64	0.59		C**O**OH	220.90	7.68	0.58
	CO**OH**	117.34	-8.21	0.46		CO**OH**	118.11	-8.19	0.46
dmpn-Mg_2(dobpdc)	M-**O**CO	165.56	-7.56	0.96	dmpn-Zn_2(dobpdc)	M-**O**CO	164.87	-8.23	0.92
	M-OC**O**	182.68	-7.23	0.87		M-OC**O**	185.12	-7.15	0.89
	C**O**OH	229.71	7.87	0.55		C**O**OH	222.77	7.78	0.59
	CO**OH**	130.65	-8.18	0.39		CO**OH**	125.23	-8.22	0.42
ii-2-Mg_2(dobpdc)*	M-**O**CO	175.77	-7.36	0.93					
	M-OC**O**	203.04	6.62	0.98					
	C**O**OH	179.35	7.53	0.90					
	CO**OH**	136.95	-8.24	0.32					

Supplementary Table 3c.- The ^{17}O NMR parameters for carbamic acid structures. The carbamic acid pairs also show two different carbon environments and hence four different oxygen environments. This is due to them being unsymmetric with respect to the organic linker backbone. In this table COOH[a] refers to the part of the pair with the C=O bond pointing towards the backbone. The COOH[b] structure refers to the part of the pair with the C-OH pointing towards the backbone. Bold letters are used to indicate the oxygen investigated.

Compound	Site	δ_{iso} (ppm)	C_Q (MHz)	η_Q	Compound	Site	δ_{iso} (ppm)	C_Q (MHz)	η_Q
pn-Mg_2(dobpdc)	C**O**OH[a]	193.07	-6.99	0.95	mpn-Mg_2(dobpdc)	C**O**OH[a]	191.84	-7.04	0.92
	CO**OH**[a]	122.17	-8.22	0.34		CO**OH**[a]	120.16	-8.22	0.34
	C**O**OH[b]	188.07	6.79	0.94		C**O**OH[b]	185.29	6.78	0.94
	CO**OH**[b]	131.52	-8.15	0.42		CO**OH**[b]	132.22	-8.11	0.39
dmpn-Mg_2(dobpdc)	C**O**OH[a]	196.48	-6.94	0.97	dmpn--Zn_2(dobpdc)	C**O**OH[a]	194.26	-6.98	0.96
	CO**OH**[a]	123.71	-8.31	0.39		CO**OH**[a]	124.24	-8.26	0.37
	C**O**OH[b]	196.61	6.78	0.92		C**O**OH[b]	194.64	6.73	0.95
	CO**OH**[b]	133.41	-8.11	0.40		CO**OH**[b]	134.35	-8.06	0.40

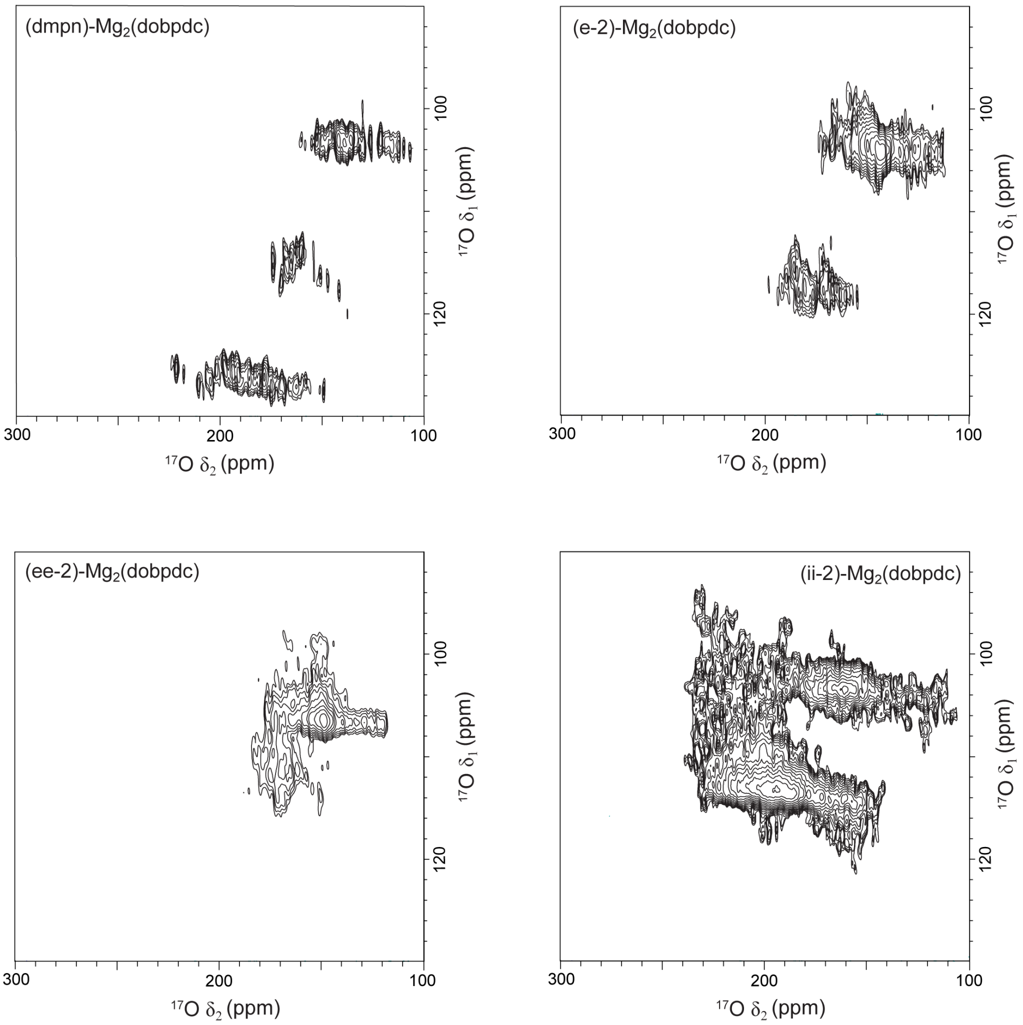

Supplementary Figure 2. - 2D MQMAS ^{17}O NMR (20.0 T, 14 KHz MAS) spectra of selected samples. Only three out of the four expected peaks for chemisorbed CO_2 were detected for (dmpn)-Mg_2(dobpdc) and (ii-2)-Mg_2(dobpdc). However, it is clear by fitting the 1D ^{17}O MAS spectra (Figure 3b, Figure 4) that an additional signal at lower chemical shifts is needed to fully deconvolute the lineshape for these materials. Although, a fourth signal is not observed in the MQMAS spectrum, likely owing to the low efficiency of multiple quantum excitation for nuclei with large quadrupolar couplings, this signal can be clearly resolved in the ^{17}O MAS spectrum at 20.0 T and 23.5 T (Figure 3, SI Figure S3, Figure 4a, SI Figure S7). In all cases, peaks from physisorbed CO_2 are not observed in the MQMAS spectra as these species are likely to have negligible quadrupolar couplings.

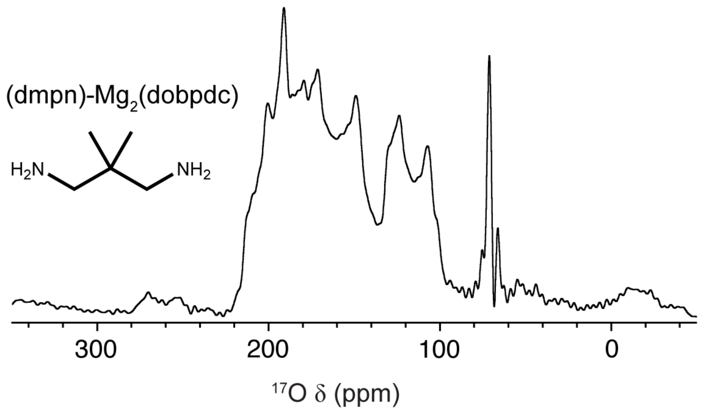

Supplementary Figure 3. - The ^{17}O NMR spectra of CO_2-dosed (dmpn)-Mg_2(dobpdc). The spectrum was taken at 23.5 T with a 20 kHz MAS rate for an independent sample to that shown in the main text at 20.0 T.

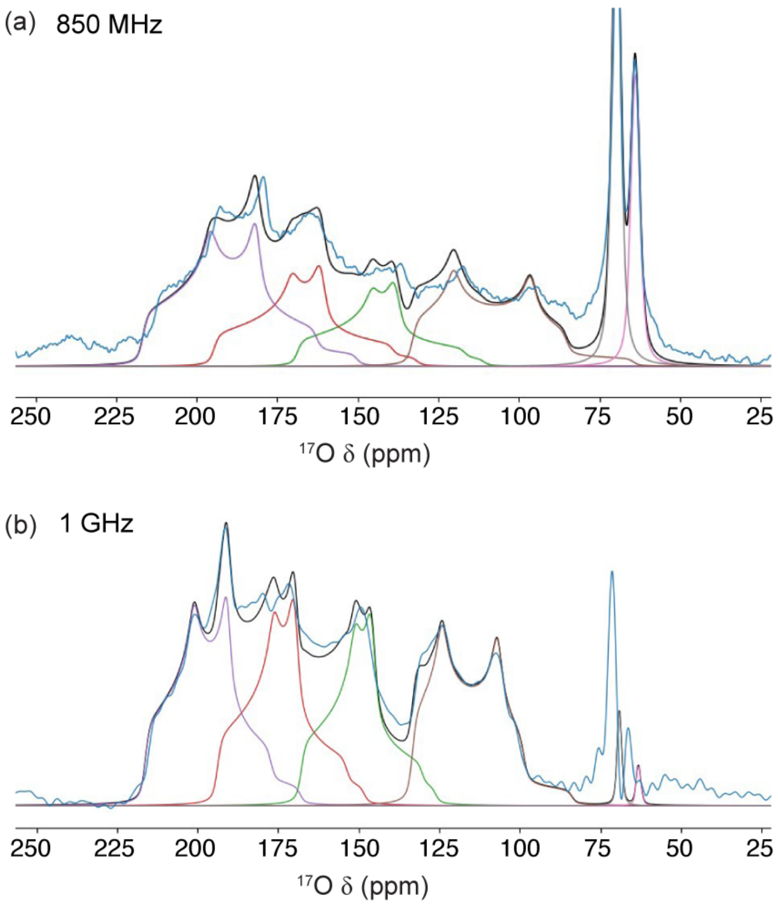

Supplementary Figure 4. - Simultaneous two field fitting of the ^{17}O NMR spectra of (dmpn)-Mg_2(dobpdc) using the program ssNake.[2] a) The resulting fit for the ^{17}O NMR data obtained at 850 MHz (20.0 T, 14kHz MAS). b) The resulting fit for the ^{17}O NMR data obtained at 1 GHz (23.5 T, 20.0 kHz). The peaks corresponding to physisorbed CO_2 were included in the fit to account for the lower shift intensities in the 850 MHz spectrum. This was done assuming two different environments for physisorbed CO_2. This fit did not account for the physisorbed peaks seen in the 1 GHz spectra indicating that other factors beyond field dependence are needed to describe the physisorbed peaks seen.

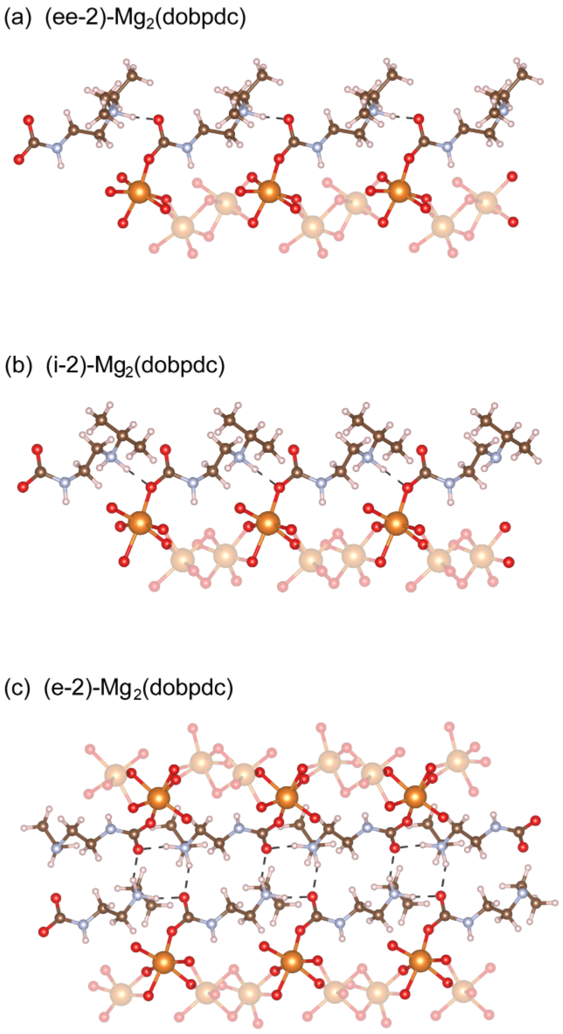

Supplementary Figure 5. - Schematics of the DFT optimised ammonium carbamate chain structures for CO_2-dosed (diamine)-Mg_2(dobpdc) samples. a) (ee-2)-Mg_2(dobpdc)–CO_2 showing H-bonding to the "free" oxygen, b) (i-2)-Mg_2(dobpdc)–CO_2 showing H-bonding to the "inserted" oxygen, and c) (e-2)-Mg_2(dobpdc)–CO_2 showing H bonding to the "free"" oxygen and H-bonding between adjacent carbamate chains.

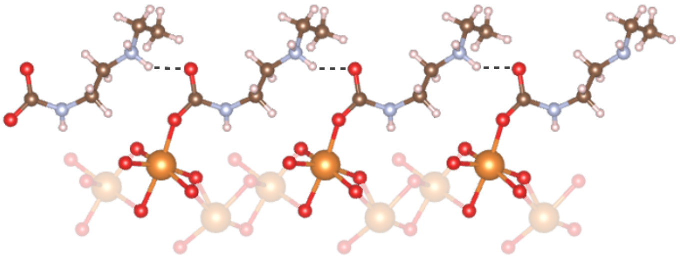

Supplementary Figure 6. The alternative structure for CO_2 dosed (e-2)-Mg_2(dobpdc) in which hydrogen bonding is taking place to the "free" oxygen. Upon geometry optimisation, the hydrogen-bonded proton moved resulting in a structure in which the CO_2 was present as carbamic acid.

Supplementary Table 4. The experimental and DFT calculated NMR parameters for CO_2 dosed (e-2)-Mg_2(dobpdc). In addition to the DFT structure shown in the main text (Figure 4b, Table 1), two alternative structures were investigated. The carbamic acid structure is the structure resulting from geometry optimisation of the structure Figure S6. The second structure is the same structure as in Figure S6, *i.e.*, without geometry optimisation. This second structure had the same hydrogen bonding arrangement as CO_2 dosed (ee-2)-Mg_2(dobpdc) (Figure S5a) and show a better fit between DFT calculated and experimental parameters. Bold letters are used to indicate the oxygen investigated.

Compound	DFT structure	δ ^{17}O (ppm)	C_Q (MHz)	η_Q
		Experiment (DFT)	Experiment (DFT)	Experiment (DFT)
(e-2)–Mg_2(dobpdc)	Carbamic acid	M-**O**CO: 171 (175)	6.9 (7.3)	1.0 (1.0)
		M-OC**O**: 197 (135)	6.3 (7.9)	0.9 (0.4)
(e-2)–Mg_2(dobpdc)	Figure S6	M-**O**CO: 171 (176)	6.9 (6.9)	1.0 (0.8)
	without geometry optimisation	M-OC**O**: 197 (188)	6.3 (7.3)	0.9 (0.8)

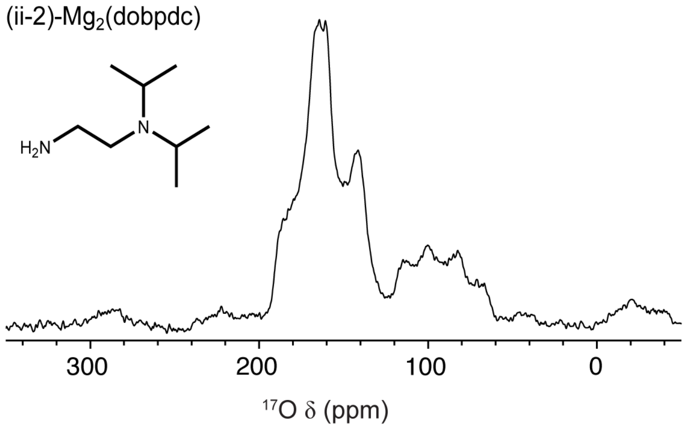

Supplementary Figure 7. **- The ^{17}O NMR spectra of CO_2-dosed (ii-2)-Mg_2(dobpdc).** The spectrum was taken at 20.0 T with a 14 kHz MAS rate for an independent sample to that shown in the main text at 23.5 T.

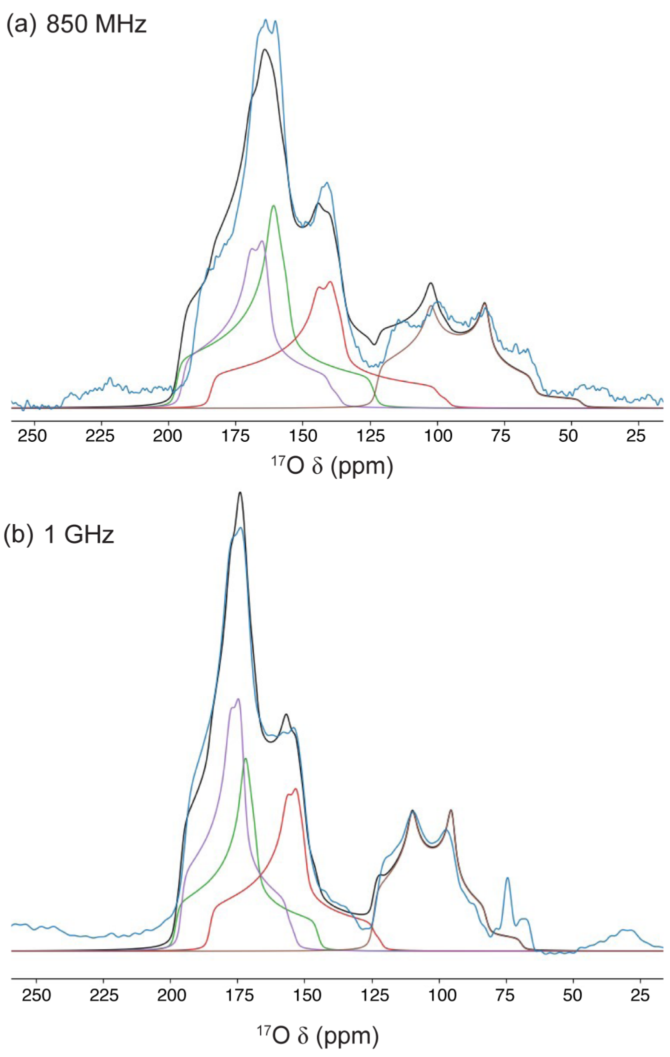

Supplementary Figure 8. - Simultaneous two field fitting of the ^{17}O NMR spectra of (ii-2)-Mg_2(dobpdc) using the program ssNake.[2] a) The resulting fit for the ^{17}O NMR data obtained at 850 MHz (20.0 T, 14kHz MAS). b) The resulting fit for the ^{17}O NMR data obtained at 1 GHz (23.5 T, 20.0 kHz).

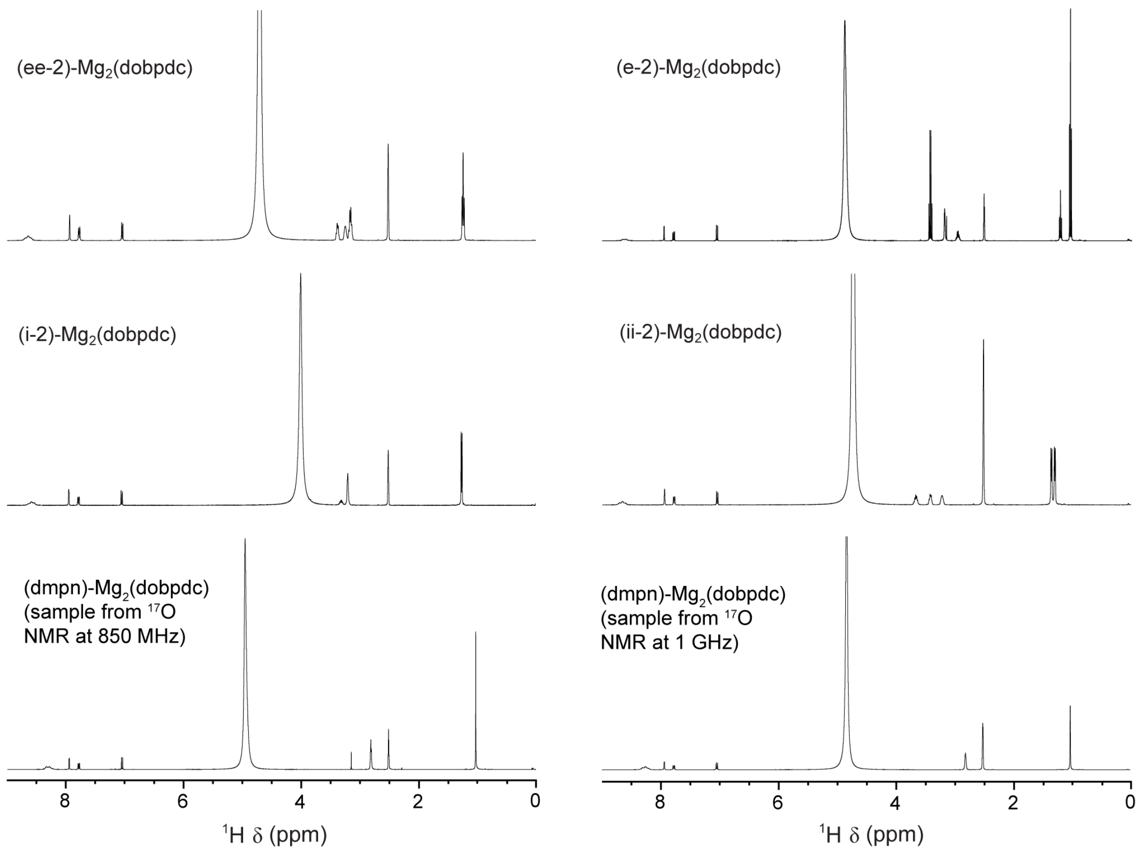

Supplementary Figure 9. - Quantitative solution ^{1}H NMR (400 MHz) spectra of acid-digested (diamine)-Mg_2(dobpdc) samples.

Supplementary Table 5. - Stoichiometries and diamine loadings of amine-functionalised frameworks determined by ^{1}H solution-state NMR of acid-digested samples.

Diamine	Formula	Diamine loading
i-2	Mg_2(dobpdc)(i-2)$_{1.92}$	96%
e-2	Mg_2(dobpdc)(e-2)$_{2.09}$	104%
ee-2	Mg_2(dobpdc)(ee-2)$_{1.89}$	95%
dmpn – 20.0 T sample	Mg_2(dobpdc)(dmpn)$_{2.02}$	101%
dmpn – 23.5 T sample	Mg_2(dobpdc)(dmpn)$_{2.00}$	100%
ii-2 – 20.0 T sample	Mg_2(dobpdc)(ii-2)$_{1.88}$	94%
ii-2 – 23.5 T sample	Mg_2(dobpdc)(ii-2)$_{2.04}$	102%

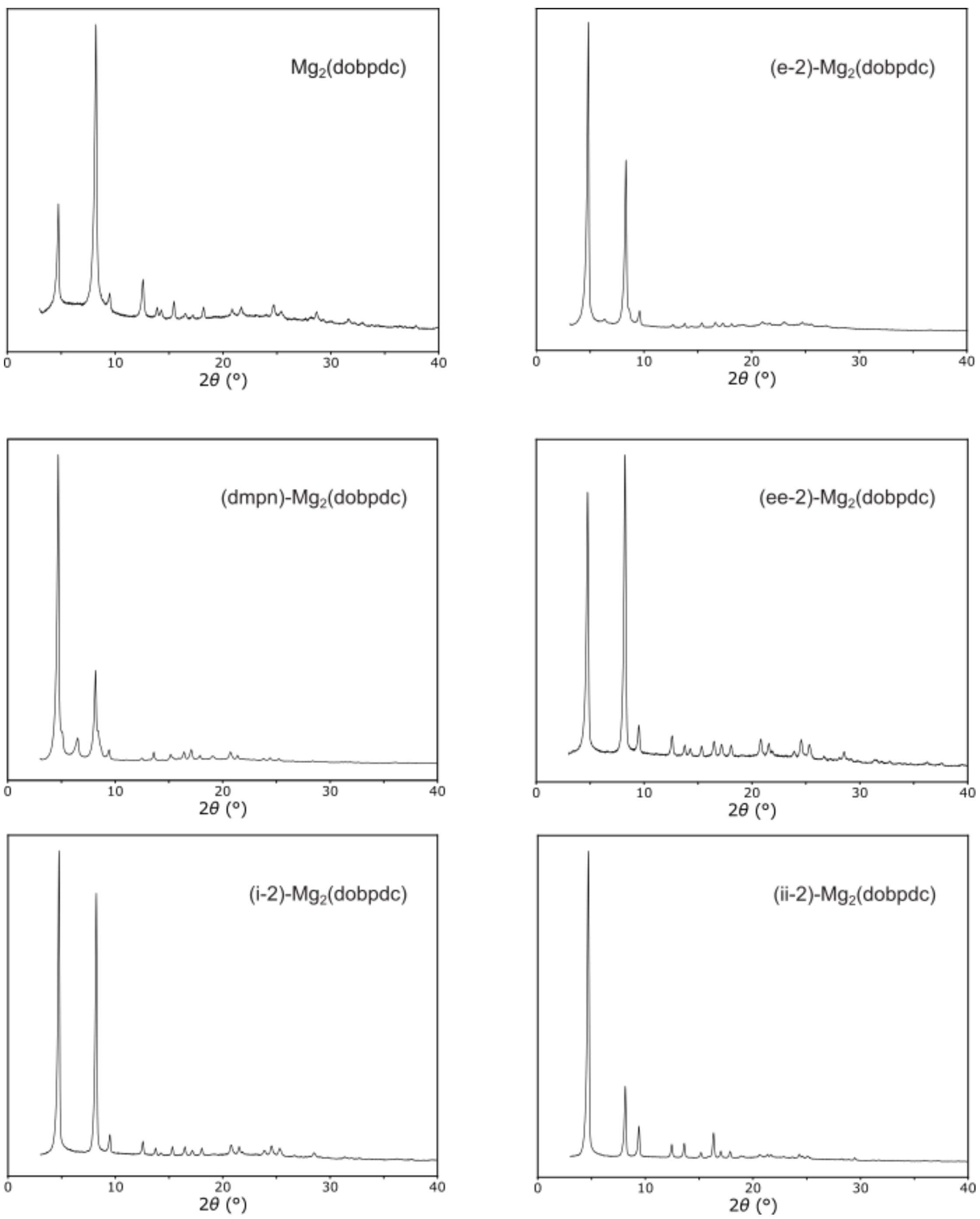

Supplementary Figure 10. - PXRD patterns of Mg_2(dobpdc) and the investigated (diamine)-Mg_2(dobpdc) samples. The PXRD was taken using a Malvern Panalytical Empyrean instrument equipped with an X'celerator Scientific detector, using non-monochromated Cu Kα radiation (λ = 1.5418 Å). Each sample was placed in a glass sample holder and measured in reflection geometry with sample spinning. The data was collected at room temperature over a 2θ range of 2-40 °, with an effective step size of 0.01 – 0.02 ° and a total collection time of 45 min.

Supplementary Table 6. – Basic characterisation of amine-grafted silicas. Elemental analysis and CO_2 uptake by thermogravimetric analysis experiments in 15% CO_2/N_2.

Sample name	Organic content (%)	Nitrogen content (mmol/g)	CO_2 Adsorption (mmol/g)			CO_2/N		
			25 °C	50 °C	75 °C	25 °C	50 °C	75 °C
**SBA-15**	0	0	0.12	0.05	0.02	-	-	-
**Tri-Si**	30.43	6.34	1.35	1.66	1.50	0.21	0.26	0.24
**Pr-Si**	20.83	3.59	1.43	1.31	1.11	0.40	0.36	0.31

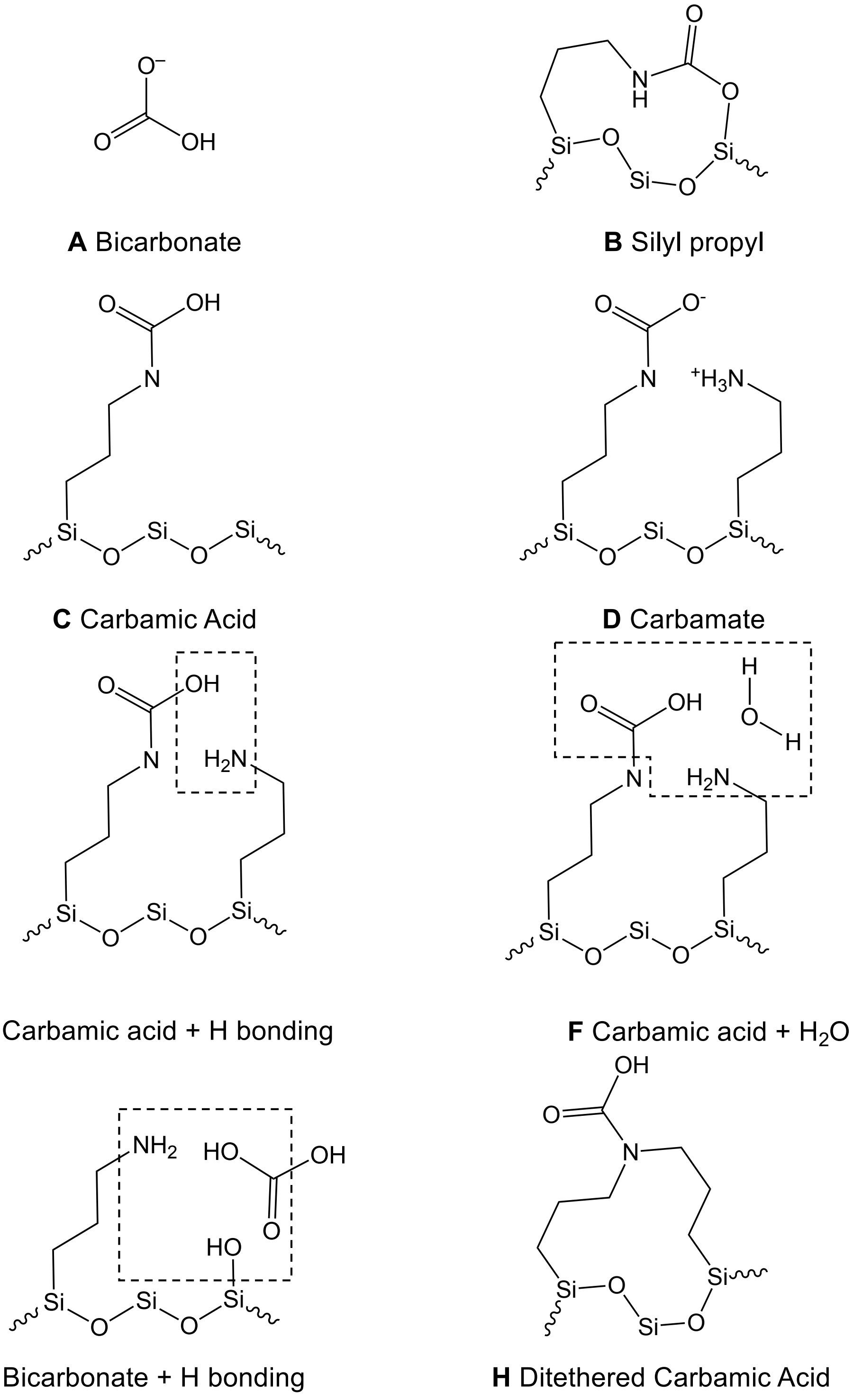

Supplementary Figure 11. – Amine-grafted silica structures investigated by cluster calculations in Gaussian software.

Supplementary Table 7. – The ^{17}O NMR chemical shifts calculated for amine-grafted silica structures shown in Figure S11.

Bold letters are used to indicate the oxygen investigated.

	Structure	O site	^{17}O δ_{calc} (ppm)	^{17}O C_Q /MHz	^{17}O η_Q	^{17}O $\delta_{calc,obs}$ (ppm)
**A**	Bicarbonate	C=**O**	178.2	7.8	0.75	154.4
		C-**O**	174.3	8.4	0.56	149.1
		C-**O**H	136.7	9.6	0.78	100.2
**B**	Silyl Propyl	C-**O**-Si	138.1	9.3	0.13	110.0
		C=**O**	212.1	8.4	0.50	187.4
**C**	Carbamic Acid	C=**O**	243.9	8.7	0.39	218.1
		C-**O**H	120.7	9.8	1.00	79.3
**D**	Carbamate	C-**O**	187.1	7.7	0.98	161.9
		C=**O**	191.1	7.6	0.69	169.1
**E**	Carbamic Acid + H bonding	C-**O**H	117.8	10.1	1.00	73.7
		C=**O**	208.2	8.2	0.62	183.3
**F**	Carbamic Acid +H_2O	C-**O**H	118.8	10.0	1.00	74.8
		C=**O**	205.3	8.3	0.64	179.9
**G**	Bicarbonate + H bonding	C-**O**H	120.0	9.9	1.00	77.6
		C-**O**H-NH2	140.5	8.7	1.00	107.8
		C=**O**	229.9	8.2	0.39	206.8
**H**	Ditethered Carbamic Acid	C-**O**H	98.4	10.0	1.00	54.9
		C=**O**	251.4	8.8	0.24	225.8

Supplementary References

(1) Kim, E. J.; Siegelman, R. L.; Jiang, H. Z. H.; Forse, A. C.; Lee, J. H.; Martell, J. D.; Milner, P. J.; Falkowski, J. M.; Neaton, J. B.; Reimer, J. A.; Weston, S. C.; Long, J. R. Cooperative Carbon Capture and Steam Regeneration with Tetraamine-Appended Metal-Organic Frameworks. *Science* **2020**, *369* (6502). https://doi.org/10.1126/science.abb3976.

(2) van Meerten, S. G. J.; Franssen, W. M. J.; Kentgens, A. P. M. SsNake: A Cross-Platform Open-Source NMR Data Processing and Fitting Application. *Journal of Magnetic Resonance* **2019**, *301*, 56–66. https://doi.org/10.1016/J.JMR.2019.02.006.

(3) Martell, J. D.; Porter-Zasada, L. B.; Forse, A. C.; Siegelman, R. L.; Gonzalez, M. I.; Oktawiec, J.; Runčevski, T.; Xu, J.; Srebro-Hooper, M.; Milner, P. J.; Colwell, K. A.; Autschbach, J.; Reimer, J. A.; Long, J. R. Enantioselective Recognition of Ammonium Carbamates in a Chiral Metal-Organic Framework. *Journal of the American Chemical Society* **2017**, *139* (44). https://doi.org/10.1021/jacs.7b09983.

(4) Čendak, T.; Sequeira, L.; Sardo, M.; Valente, A.; Pinto, M. L.; Mafra, L. Detecting Proton Transfer in CO2 Species Chemisorbed on Amine-Modified Mesoporous Silicas by Using 13C NMR Chemical Shift Anisotropy and Smart Control of Amine Surface Density. *Chemistry - A European Journal* **2018**, *24* (40). https://doi.org/10.1002/chem.201800930.
